# Blinding Is Seeing: A Single-Centre Study Into the Viability of Auto-Injectors for Blinded-Drug Administration in Randomised Controlled Trials

**DOI:** 10.7759/cureus.44244

**Published:** 2023-08-28

**Authors:** Vishal Aggarwal, Jorg Taubel, Ulrike Lorch, Thomas York

**Affiliations:** 1 Clinical Research, Richmond Pharmacology Ltd, London, GBR; 2 Cardiology, Richmond Pharmacology Ltd, London, GBR

**Keywords:** medical device technology, imp, injection, double blind, clinical trial, auto-injector

## Abstract

Objective

The aim of the study was to assess the viability of auto-injector systems (A-INJ) for preserving investigator blinding in randomized controlled trials (RCT).

Background

Blinding refers to the concealment of group allocation from one or more individuals involved in a clinical research study. In the dosing of subcutaneous (SC) and intramuscular (IM) investigational medicinal products (IMP), specific challenges arise in maintaining investigator blinding. These challenges primarily involve the active injectate's viscosity and visual appearance (colour/translucency) in comparison to the placebo. Existing methods to control these issues are not perfect. Common approaches include using unblinded investigators or applying films or additives to make the active and placebo injectates appear similar.

Method

A single-centre experimental and descriptive study was carried out to compare the use of an A-INJ (Owen Mumford, Autoject 2) with the use of a conventional syringe (CS) in delivering a 1 ml dose of both placebo and reference IMP. The percentage delivery of the injectate was compared between the A-INJ IMP and placebo groups. Additionally, eight trained research physicians serving as investigators recorded their assessments of safety and effectiveness after performing serial injections with the A-INJ into a human-tissue analogue.

Results

Overall, a mean of 95.38% of 1ml placebo injectate was released from the A-INJ, compared to 96.00% from the CS. A total of 94.715% of 1 ml IMP injectate was released from the A-INJ, as opposed to 94.74% from the CS. Independent t-test analyses showed no statistical significance between the experimental arms. The mean administration time was 8.5 seconds. Investigators were unable to differentiate between the two solutions when using the A-INJ. There were no recorded concerns about investigators becoming unblinded, which stands in contrast to concerns associated with using the CS.

Conclusion

In assessing the viability of A-INJ use in RCTs, we noted a marked improvement when blinding was used. A-INJ systems effectively administer both placebo and active injectates, thereby maintaining the benefit of blinding without the need to alter the placebo through the addition of colourants or viscosity additives. While audio cues from the A-INJ and the time required to administer the injectate pose challenges, solutions are suggested. Although our findings are preliminary, they add to the existing literature on the advantages of A-INJs for administering injectable compounds and offer new perspectives on their utility in RCTs.

## Introduction

The need for blinding

As the foundation of randomised controlled trials (RCTs), data integrity is crucial, which calls into question commercial practices, data integrity, and trial results. Cohort randomisation and blinding are employed to mitigate such concerns. Although randomisation minimises the selection bias, reducing the likelihood of prognostic differences between intervention groups, its use does not prevent the biased assessment of outcomes. Blinding is crucial for maintaining study integrity and evaluating the intervention's objective performance.

Within the clinical trial space, rigorous, meticulous RCTs provide the best estimate of an intervention’s impact. Often in the form of an injectate, this has historically been administered through the use of a conventional syringe (CS). With precise demarcations and various needle attachments, its use relies on the localisation of an injection site followed by controlled insertion and slow plunger depression. The visible barrel, exposed needle, and tactile plunger provide opportunities for an investigator to dynamically ascertain data pertaining to any given injection in progress, which could ultimately lead to inadvertent unblinding.

When determining the impact of therapies, investigational medicinal products (IMPs) can sometimes exert effects beyond those expected. A non-specific effect is an outcome that does not arise according to an intended mechanism of action. It can be a response to a placebo or a reflection of spontaneous events, either causal or correlated to administration. A placebo can take several tangible and intangible forms. The response to a placebo can be either positive for the outcome of interest, known as the placebo effect, or negative for the outcome of interest, known as the nocebo effect. Both ultimately shape the investigational expectancy of the results. Evidence suggests that expectations induce bias. In a double-blind sham surgery trial investigating a new surgical transplant technique for treating Parkinson’s disease, sham and genuine surgical interventions were equally effective. However, participants who thought they received the transplant reported a better quality of life. The perceived benefit from the treatment seemingly triggered positive expectations. It has also been argued that positive reporting was in response to guided questioning, which is a bias unto itself.

To control for study expectations, the double-blind RCT design is commonly used to examine a novel intervention for its specific effects. However, its origin harks back to the decidedly uncommon court of French nobility. In 1784, Louis XVI appointed the 'Royal Commission on Animal Magnetism' to investigate Charles d'Eslon's theory of 'fluide magnétique.' According to this theory, animate beings contained a bodily, magnetic fluid influenced by astronomical movements, which shaped one's health and emotions. The commission, which included Benjamin Franklin, determined that physically blindfolding both investigators and subjects would allow 'sham' and 'genuine' procedures to be discerned. As suggested by its conspicuous absence from contemporary medical practice, the theory of animal magnetism was quickly discounted. However, the rationale for blinding, embraced by the enlightened minds of the Paris Faculty of Medicine, has enjoyed far greater longevity.

When neither the participants nor the investigators are aware of the intervention or placebo, expectations are balanced across groups, regardless of celestial involvement. Double-blinding when properly implemented makes groups comparable so that specific and non-specific treatment effects can be ascertained with less potential for bias and expectation [6].

There is evidence of imperfect double-blinding in many reportedly double-blinded RCTs. Several factors can undermine the double-blinding methodology, including poor randomisation methods, inadequate concealment of allocation, and the use of a placebo that is distinguishable from the intervention [7]. In the context of syringe-administered therapy, this is typically due to the following.

Solution Colour

A disparity in the visual appearance of the two solutions can be sufficient to allow differentiation between the IMP and the placebo, a consequence of the wavelengths of light absorbed by their constituents. Interestingly, there is a distinct lack of reporting on issues pertaining to blinding methods, yet common measures to address these problems have emerged. One method involves applying a coloured translucent film to the syringe barrel, allowing investigators to monitor the rate and completion of delivery while standardizing the appearance of the solution. Another approach has been to color-match the placebo to the IMP using an additive [8]. While these methods are viable in a clinical setting, it is worth noting that they raise concerns about the inherent nature of the placebo and its potential for influencing results.

Solution Viscosity

Compared to conventional placebos like saline or sterile water, the IMP often has a noticeably different viscosity. This difference is detectable due to the haptic feedback experienced when depressing the syringe plunger; the viscosity alters the force needed or the total time taken to administer the IMP. As with colourants, the current solution involves using additives to modify and match the viscosity. It should be noted that the same concerns about unnecessary injection risk and data integrity remain, and formulations of placebos are often poorly documented in clinical study protocols. While there are widely accepted conventions on the characteristics of an appropriate placebo [9], regulatory agencies provide scant guidance on this matter.

An autoinjector serves as an alternative to the CS, designed for the administration of pre-measured doses of medication. With a spring-loaded mechanism, the autoinjector ensures consistent and precise delivery of medication through a concealed chamber and needle.

Current injection methods and the risk of unblinding

There are several methods for delivering drugs to various tissue levels, and recent advances in drug transport dynamics may guide further improvements to the performance of current and alternative injection technologies [10].

Two 2016 studies by Hill et al. outlined the mechanical influences of IM injection methods and tissue on drug permeability in an animal model, with the advantages of muscle mass parameters closely resembling human skeletal muscle [11]. The autoinjector system (A-INJ) provided higher peak volumes of injectate without exaggerated surface plebs, indicating a greater degree of dispersion compared with manual syringe delivery. Given the ability to adjust the exposed, and therefore, penetrating needle length to match the tissue thickness, this highlights another advantage of A-INJ design versus CS. In the second study, the EpiPen A-INJ resulted in larger dispersion volumes and higher initial dispersion ratios, which decreased rapidly over time, suggesting a greater rate of uptake of injectate than other A-INJs. The differences in dispersion and uptake of injectate were likely the result of different functional characteristics of the delivery systems and demonstrated the functional characteristics of A-INJ for delivering IM injectate.

Four major types of encapsulated mechanoreceptors are specialized to provide information to the central nervous system about touch, pressure, vibration, and cutaneous tension: Meissner’s corpuscles, Pacinian corpuscles, Merkel’s disks, and Ruffini’s corpuscles. These are collectively referred to as low-threshold mechanoreceptors because even weak mechanical stimulation of the skin induces them to produce action potentials and subsequent outcomes, including sensation, through large AB myelinated axons, ensuring the rapid central transmission of tactile information [12]. A psychophysics study by Skedung et al. in 2013 showed that across 16 artificially created surfaces with 'wrinkle' wavelengths ranging from 300 nm to 90 mm, and amplitudes between 7 nm and 4.5 mm, the human finger was able to discern tactile differences of 10 nm, confirming tactile discrimination to a nanoscale degree [13]. While not the primary focus of our study, plunger force modifiers provide unique insight into the variability of injection metrics that could ultimately unblind a study, given the human hand’s low threshold for tactile discrimination.

If an RCT is not adequately double-blinded, expectations arise and disrupt unbiased treatment arms, affecting data integrity. Therefore, we propose a novel method of using A-INJ to deliver IMP and placebo during RCTs, mitigating application bias to sustain blinding and improve trial accuracy and objective outcomes [14].

The use of an A-INJ as opposed to CS in RCTs that predominantly employ the SC administration method requires exploration but is beyond the scope of our current investigation. In a 2015 review by Jin et al., significant variability in the pharmacodynamic effects of SC versus IM injections was demonstrated, due to a variety of physical-pharmaceutical factors such as compound, needle gauge, etc., and physiological differences such as injection site, metabolic pathologies, and adipose thickness [15]. We acknowledge that the effect of injection methods on pharmacodynamics presents further research opportunities.

## Materials and methods

Study design

Our single-centre experimental and descriptive study assessed A-INJ against CS for the administration of injectate. No ethical approval was required. The testing had three primary aims: (1) to determine the full-dose viability of A-INJ-based active compound 'IMP' delivery; (2) to perform usability testing in skeletal muscle analogues (SMAs); and (3) to determine the effectiveness of blinding, specifically assessing whether there was a lack of identifiable haptic, visual, or other cues between IMP and placebo when using the A-INJ.

Protocol-Part A: Quantitative Data Collection on the Differences Between CS and A-INJ

An empty control syringe (CS) and a 12.7 mm needle were carefully weighed without the needle cap and were denoted as point 'A'. A precise 1 ml solution was drawn into the syringe, and then the filled syringe and needle were weighed again without the needle cap, referred to as point 'B.' To ensure accuracy, any excess air in the syringe was meticulously removed by holding it upright, as is typical within clinical practice. Weight measurements at points A and B allowed for the calculation of the injectate by subtraction: A - B.

An empty beaker was placed on a scale and tared. The injectate was expelled from the syringe into the empty beaker, and the weight of the released injectate was recorded through the change in the beaker's total mass. Any potential loss of product during the release was also noted. This procedure, from weighing to release, was performed twice for both the placebo and active compounds, for both the CS and A-INJ; a total of four CS and four A-INJ full data sets were obtained.

Protocol-Part B: Qualitative Data Collection on Practical Use Between CS and A-INJ

Skinless chicken breasts of comparable densities were used as SMAs. Before the injection phase, the SMA samples were left at room temperature for 60 minutes to equalise. Following the temperature equalisation, a 1 ml placebo solution was drawn into the CS for administration. The injectate from the syringe was systematically administered into the SMA samples, using industry-standard IM injection techniques. A new injection site was used for each of the three injections per SMA, approximately 1 inch from the previous insertion point. This process was then repeated using A-INJ in place of CS on a second chicken breast per investigator. Three CS and three A-INJ injections across two SMAs per investigator totalled thirty administrations.

During the process, investigators self-reported observations and any pertinent details regarding ease of use or concerns. These recordings were written at the point of declaration to ensure accurate capture and to avoid recall subjectivity.

Assessment

The accuracy of the injected volume was evaluated by the gravimetric method using the Sartorius 0.0001 mg balance. The weight of the injection system was recorded before and after each injection. Full dosing was considered complete if ≥95.0% of the injectate was dispensed, as is standard practice in our unit. Injectate leakage was accounted for through the closed containment system in which the testing took place and was again assessed through the gravimetric method. The injected volume was calculated based on the weight of the injection systems before and after injection, accounting for losses where applicable. Qualitative data pertaining to CS and A-INJ efficacy and safety were assessed through investigator questionnaires following injection into SMA.

## Results

**Figure 1:**
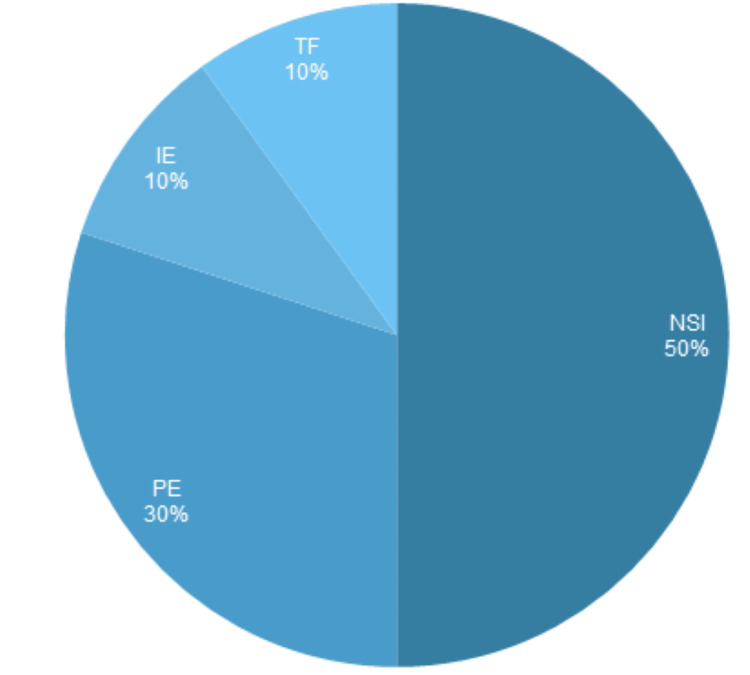
Use concerns during CS use from syringe-competent physicians. NSI, needle stick injury; PE, premature ejection; TF, technical failure; IE, incomplete ejection; CS, conventional syringe.

Our control investigation examined the loading and use of CS by syringe-competent physicians. Five physicians completed a questionnaire about using the CS, and while full dosing was achieved-in which >95.0% of the injectate was discharged without measurable loss-concerns about use were raised on all accounts (Table 1). The risk of NSI was highlighted by all investigators, constituting 50% of total concerns raised (Figure 1). This is not dissimilar to findings derived from NSI reporting across multiple healthcare professional groups [15, 16]. The potential for injectate loss was also mentioned but requires a larger sample size and more precise assessment methods before claims of substantiality and pharmacoeconomic implications can be determined.

**Table 1:** Physician self-reporting of CS injection into SMAs NSI, needle stick injury; PE, premature ejection; TF, technical failure; IE, incomplete ejection; CS, conventional syringe; SMA, skeletal muscle analogue.

Placebo	Ease of Use	Comments
Investigator 1	Y	Risk of NSI, high chance of PE
Investigator 2	Y	Risk of NSI, chance of PE
Investigator 3	Y	Risk of NSI, needle bent during application/TF
Investigator 4	Y	Risk of NSI, chance of PE
Investigator 5	Y	Risk of NSI, risk of IE

Data collected for Part A of the protocol using an A-INJ with placebo are outlined in Table 2, while comparative data for the active compound are presented in Table 3. Statistical analysis in Appendix reveals that the two A-INJ samples loaded with active compound (M = 94.15, SD = 2.55) showed no statistically significant difference in volume dispersion compared to the two samples in the placebo group (M = 95.38, SD = 0.71), t(2) = 0.96, p = .05.

**Table 2:** Observed metrics for autoinjector loaded with 1 ml placebo

Placebo	Empty syringe weight (A) [g]	Filled syringe weight (B) [g]	Injectate weight [(B)-(A)] [g]	Injectate released into beaker [g]	Injectate released into beaker [%]	Loss [g]	Full dosing achieved (Y/N)
Autoinjector I	2.2257	3.2613	1.0356	0.9939	95.97	0.0417	Y
Autoinjector II	2.2182	3.2709	1.0527	0.9978	94.78	0.0549	Y

**Table 3:** Observed metrics for autoinjector loaded with 1 ml active compound

Active	Empty syringe weight (A) [g]	Filled syringe weight (B) [g]	Injectate weight [(B)-(A)] [g]	Injectate released into beaker [g]	Injectate released into beaker [%]	Loss [g]	Full dosing achieved (Y/N)
Autoinjector III	2.2015	3.3417	1.1402	1.0606	93.02	0.0796	Y
Autoinjector IV	2.2441	3.3662	1.1221	1.0691	95.28	0.053	Y

Data collected for Part A of the protocol using CS with placebo are detailed in Table 4, and the comparative data for the active compound are provided in Table 5. Statistical analysis in Appendix shows that the two CS samples loaded with active compound (M = 94.74, SD = 0.45) also demonstrated no statistical significance in volume dispersion when compared to the two samples in the placebo group (M = 96.00, SD = 0.20), t(2) = 2.21, p = .05. These results indicate that A-INJ is as effective as CS for utilisation in RCTs, given that the objective is to expel various fluid compounds in an effective manner.

**Table 4:** Observed metrics for a conventional syringe loaded with 1 ml placebo

Placebo	Empty syringe weight (A) [g]	Filled syringe weight (B) [g]	Injectate weight [(B)-(A)] [g]	Injectate released into beaker [g]	Injectate released into beaker [%]	Loss [g]	Full dosing achieved (Y/N)
CS I	2.2194	3.2603	1.0409	0.9959	95.68	0.045	Y
CS II	2.245	3.2803	1.0353	0.9972	96.32	0.0381	Y

**Table 5:** Observed metrics for a conventional syringe loaded with 1 ml active compound

Active	Empty syringe weight (A) [g]	Filled syringe weight (B) [g]	Injectate weight [(B)-(A)] [g]	Injectate released into beaker [g]	Injectate released into beaker [%]	Loss [g]	Full dosing achieved (Y/N)
CS III	2.2167	3.2617	1.0450	0.9949	95.21	0.0501	Y
CS IV	2.2201	3.2802	1.0601	0.9992	94.26	0.0609	Y

Additional findings emphasised the functional characteristics of the injection delivery systems and their impact on investigator perception during injection into SMAs, as summarised in Table 6. Although investigators were familiar with CS, they reported various functional limitations as listed in Table 1. Notably, no instances of needlestick injuries (NSI) occurred, but the risk remained a prevalent concern, highlighting reservations about the existing technology. Interestingly, all investigators found the A-INJ easy to use; however, during the thirty A-INJ administrations, a single instance of needle bending was observed during the draw-up process. When identifying disparities between placebo and active injections, several performance metrics were considered, with syringe haptics predicted to be the most telling. 

**Table 6:** Physician self-reporting during autoinjector use into skeletal muscle analogues

PLACEBO	Ease of Use	Comments
Investigator 1	Y	Needle of internal syringes bends during draw-up
Investigator 2	Y	-
Investigator 3	Y	-
Investigator 4	Y	-
Investigator 5	Y	-

## Discussion

To the best of our knowledge, this study marks the first documented formal comparison between autoinjectors (A-INJ) and conventional syringes (CS) when used by physicians in the context of randomised controlled trials (RCTs). The primary aims were to evaluate performance, safety, and feasibility, specifically in the setting of RCTs.

In 1982, Nelson and Houtchens investigated the effects of operator variability on thermodilution cardiac output measurements. A cohort of physicians and nurses performed a series of manual and automatic injections using a gas pneumatic injector in a simulated clinical setting. The data indicated significant variability in parameters such as injection time, injectate flow rate, and consistency of injections during manual procedures. In contrast, there was minimal variability in these metrics during automatic injections [17]. When other variables are adequately controlled, automatic injectors enhance the precision of cardiac output measurements by maintaining consistency in the injections and minimising operator-induced variables. These findings can reasonably be extrapolated to RCTs to suggest that autoinjectors may improve drug delivery, reduce administration errors, and enhance data integrity.

Irrespective of IMP or placebo, a CS requires the user to apply a force sufficient to deliver the injectate. The extent of this force depends on several parameters, including user dexterity, fluid viscosity, needle length and gauge, friction between the syringe plunger and barrel, the cross-sectional area of the syringe plunger, and plunger displacement [18]. As per the findings of Skedung et al., tactile differentiation of such factors is likely, but confirmatory nerve conduction and pressure studies would need to be performed [9]. A-INJ provides a consistent force profile to push the drug out of the syringe [8]. The internal spring is compressed prior to activation and is released either by a button press or by applying pressure to the needle end of the A-INJ, depending on the design. The spring force is designed to be sufficient to deliver the drug, with a consistent activation energy, independent of the syringe’s contents [10].

A variety of A-INJ SC cannula and spring force combinations were assessed by Rini et al. in 2022, highlighting various 2 ml injection durations across viscosities, with ultra-thin-walled syringes enabling faster rates. Two millilitres represent the largest single volume typically administered in an RCT, and while marginally less time for a needle in situ provides fewer opportunities for infection and tissue insult, the real-world, clinical effects are arguably negligible [19]. In an RCT setting, however, where optimisation of the protocol boasts safety, resource, and subsequently cost implications, mitigating the human factors during large-scale, large-volume A-INJ administration is worthy of exploration.

The audible nature of a spring 'firing' was a point of observation, posing a stark difference to the silent CS. It should be noted that this initial sound poses no means of unblinding, but a completion sound, present on some A-INJ models, could uncover the underlying compound. A less viscous, i.e., placebo, would complete the injection faster, thereby triggering the completion sound earlier. The device chosen for the study was employed for its fortunate lack of a completion sound, and while the duration of injection still poses a method of unblinding, this was controlled by holding the A-INJ against the injectable surface for a predetermined period of time; long enough to facilitate the lengthier push of either the placebo or the active injectate, determined by the already unblinded pharmacists during dosing setup [20].

Whilst providing a proof-of-concept for the implementation of A-INJ in RCT, it is clear that further work is needed. Given the predominance of observational measures, a concerted effort against inducing biases was made; however, we acknowledge that subconscious bias will require a more complete study design to eradicate fully. Our quantitative measures are deemed to be reliable. Confounding factors, where possible, were controlled through a climate-controlled testing environment and a clinical trials-grade pharmaceutical manufacturing laboratory for placebo and active injectate constitution.

With the small sample size and the limited supply of A-INJ for the purposes of such a trial, it will be necessary to conduct a prospective implementation study to assess the efficiency and efficacy of their use over CS when scaled up to typical RTC levels. In addition, whilst the upper volume for a single RCT injection is 2 ml, a reduction in the number of injections per participant would be predicated on the ability to administer a larger volume via A-INJ, should it be required. Testing for this would require assessment alongside prospective pharmacoeconomic outcome measures, which should be encouraged to outline the setup and running costs of A-INJ. Future efficacy and safety studies should also include contemporaneous investigator preference, featuring anyone likely to dose a participant, not just physicians with CS experience.

## Conclusions

The idea that using an A-INJ system is associated with quality improvement benefits is not new and has been alluded to by multiple clinical and research organisations. A-INJ systems demonstrate consistent utility in administering both placebo and IMP, while maintaining blinding without the need for colourants or viscosity additives. It should be noted that the audio cues of the A-INJ 'firing' contrast with the silent application of a manual syringe. However, with the Owen Mumford Autoject 2, the lack of a 'discharge completion' sound maintains study integrity, provided the time to discharge is controlled for. With these minor caveats accounted for, and as per established literature on the use of A-INJ versus CS across a wide range of clinical and research applications, no group, ourselves included, has identified a drawback or disadvantage to using A-INJ systems. Our results, combined with previously documented findings, present a convincing argument that the use of A-INJs improves the quality of blinding and efficiency, and should be standard practice in all double-blind RCTs with injectable IMP.

## Appendices

**Table 7:** Independent T-test comparing A-INJ placebo against active injectate A-INJ: autoinjector.

Independent T-test	Injectate released into beaker [%]		Difference (A-INJ -M)			Sq. Diff		
	1	2	1	2	M	1	2	SS
A-INJ Placebo	95.97	94.78	0.59	-0.59	95.38	0.35	0.35	0.71
A-INJ Active	93.02	95.28	-1.13	1.13	94.15	1.28	1.28	2.55

A-INJ placebo

N_1_: 2 df_1_ = N - 1 = 2 - 1 = 1 M_1_: 94.74 SS_1_: 0.45 s^2^_1_ = SS_1_/(N - 1) = 0.45/(2-1) = 0.71

A-INJ active

N_2_: 2 df_2_ = N - 1 = 2 - 1 = 1 M_2_: 94.74 SS_2_: 0.45 s^2^_2_ = SS_2_/(N - 1) = 0.45/(2-1) = 2.55

T-value

s^2^p = ((df_1_/(df_1_ + df_2_)) * s^2^_1_) + ((df_2_/(df_2_ + df_2_)) * s^2^_2_) = ((1/2) * 0.71) + ((1/2) * 2.55) = 1.63 s^2^M_1_ = s^2^p/N_1_ = 1.63/2 = 0.82 s^2^M_2_ = s^2^p/N_2_ = 1.63/2 = 0.82 t = (M_1_ - M_2_)/√(s^2^M^1^ + s_2_M_2_) = 1.22/√1.63 = 0.96

The p-value is .219334. *Not significant.*

**Table 8:** Independent T-test comparing CS placebo against active injectate. CS, conventional syringe.

Independent T-test	Injectate released into beaker [%]		Difference (CS -M)			Sq. Diff		
	1	2	1	2	M	1	2	SS
CS placebo	95.68	96.32	-0.32	0.32	96.00	0.10	0.10	0.20
CS active	95.21	94.26	0.47	-0.47	94.74	0.23	.023	0.45

CS placebo

N_1_: 2 df_1_ = N - 1 = 2 - 1 = 1 M_1_: 96 SS_1_: 0.2 s^2^_1_ = SS_1_/(N - 1) = 0.2/(2-1) = 0.2

CS active

N_2_: 2 df_2_ = N - 1 = 2 - 1 = 1 M_2_: 94.74 SS_2_: 0.45 s^2^_2_ = SS_2_/(N - 1) = 0.45/(2-1) = 0.45

T-value

s^2^p = ((df_1_/(df_1_ + df2)) * s^2^1)+ ((df_2_/(df_2_ + df_2_)) * s^2^_2_) = ((1/2) * 0.2) + ((1/2) * 0.45) = 0.33 s^2^M_1_ = s^2^p/N_1_= 0.33/2 = 0.16 s^2^M_2_ = s^2^p/N_2_ = 0.33/2 = 0.16 t = (M_1_ - M_2_)/√(s^2^_M1_ +s^2^_M2_) = 1.27/√0.33 = 2.21

The p-value is .07892. *Not significant.*
